# Vaginal microbiota diversity and paucity of *Lactobacillus* species are associated with persistent hrHPV infection in HIV negative but not in HIV positive women

**DOI:** 10.1038/s41598-020-76003-7

**Published:** 2020-11-05

**Authors:** Eileen O. Dareng, Bing Ma, Sally N. Adebamowo, Ayotunde Famooto, Jacques Ravel, Paul P. Pharoah, Clement A. Adebamowo

**Affiliations:** 1 Department of Primary Care and Public Health, University of Cambridge, Cambridge, UK; 2 Institute of Human Virology Nigeria, Abuja, Nigeria; 3 Department of Microbiology and Immunology, Institute for Genome Sciences, University of Maryland School of Medicine, Baltimore, MD USA; 4 Department of Epidemiology, Greenebaum Comprehensive Cancer Center, University of Maryland School of Medicine, Baltimore, MD USA; 5 Center for Bioethics and Research Ibadan, Ibadan, Nigeria; 6 Institute of Human Virology, University of Maryland School of Medicine, 725 West Lombard Street, Baltimore, MD 21201 USA

**Keywords:** Cancer microenvironment, Cervical cancer, Predictive markers, Epidemiology, Molecular medicine, Infection, Oncogenesis

## Abstract

The vaginal microbiota is thought to play a role in modulating risk of high-risk human papillomavirus (hrHPV) infection. We examined the relationship between the vaginal microbiota and persistent hrHPV infection in HIV-negative and HIV-positive women. We used 16S-rRNA sequencing to characterize the vaginal microbiota of two serial samples taken six months apart from 211 Nigerian women (67%, 142/211 HIV-positive and 33%, 69/211 HIV-negative) and evaluated the association between the vaginal microbiota and persistent hrHPV infection using generalized estimating equation logistic regression models and linear discriminant analysis effect size (LEfSe) algorithm to identify phylotypic biomarkers of persistent hrHPV infection. The high diversity microbiota, Community State Type IV-B, was the most prevalent in both HIV-negative (38% at baseline, 30% at the follow-up visit) and HIV-positive (27% at baseline, 35% at the follow-up visit) women. The relationship between the vaginal microbiota and persistent hrHPV was modified by HIV status. In HIV-negative women, women with *Lactobacillus* dominant microbiota had lower odds (OR: 0.35, 95% CI 0.14–0.89, *p* = 0.03) of persistent hrHPV compared to women with *Lactobacillus* deficient microbiota. While among HIV-positive women, the odds of being persistently infected with hrHPV was higher in women with *Lactobacillus* dominant microbiota (OR: 1.25, 95% CI 0.73–2.14 *p* = 0.41). This difference in effect estimates by HIV was statistically significant (p = 0.02). A high diversity vaginal microbial community with paucity of *Lactobacillus* species was associated with persistent hrHPV infection in HIV-negative women but not in HIV-positive women.

## Introduction

Cervical cancer is the third commonest cancer among women globally, with 570,000 new cases and 311,400 deaths estimated to have occurred globally in 2018^1^. Persistent infection with one of 12 high risk Human Papillomaviruses (hrHPV) is recognized as a necessary but insufficient cause of cervical intraepithelial neoplasia (CIN) and cervical cancer^2^. HPV infections are very common^3^. About 80% of sexually active women are infected with at least one HPV type at some point in their lifetimes^3^. However, around 90% of infections are cleared within 18 months, as a result of an incompletely understood host cell-mediated immune response^4^. Persistence of HPV infection leads to the accumulation of mutations in the somatic cellular genome which increases the risk of CIN and cervical cancer^5^. Interactions between host factors such as the microbiota and viral factors that facilitate persistence and carcinogenesis are not completely understood and remain an active area of research.

There is emerging evidence to suggest that the hosts’ vaginal microbiota play crucial roles in the pathogenesis of HPV infections^6,7^. The defence mechanisms against colonization of the vagina by pathogenic organisms include the presence of lactic acid producing *Lactobacillus* species^8^, an acidic environment^9,10^ and antimicrobial peptides^10,11^. Disruptions in these defence mechanisms, as could occur with high diversity vaginal microbiota, may modulate risk of viral infections such as HIV^12^ and HPV^13^. High diversity vaginal microbial communities are associated with proinflammatory responses which may directly damage the cervical epithelial barrier, thereby facilitating HPV entry to basal keratinocytes and establishment of infection^4,14^. The microbiota may also interact with cellular pathways in ways that are not yet clear, to lead to persistent, productive viral infections^14–17^.

Most 16S rRNA sequencing studies of the vaginal microbiota and hrHPV infection to date have focused on prevalent hrHPV infection and vaginal microbiome patterns^6,13,16,18–22^. Collectively, these studies showed increased risk of prevalent HPV infection in women with high diversity vaginal microbiota or with decreased relative abundance of *Lactobacillus* specie*s*^13,16,19^*.* Thus far, only one study has investigated the association between the vaginal microbiota and persistent hrHPV^21^. In that study, vaginal samples collected at one baseline visit were used to characterize the vaginal microbiota, while hrHPV persistence was determined by testing two serial samples taken 12 months apart^21^. Given that the vaginal microbiota is dynamic, cross-sectional studies of the vaginal microbiota that rely on vaginal sampling at a single time point are inadequate to fully characterize the relationship between the vaginal microbiota and persistent hrHPV^23^. Therefore, longitudinal studies with repeated vaginal microbiota assessments are warranted. Earlier work by Brotman et al. to address this knowledge gap involved extensive sampling of the vaginal microbiota and repeated HPV testing. However, that study was limited by the lack of data on high risk HPV^24^.

We previously reported an association between prevalent hrHPV and decreased relative abundance of *Lactobacillus* species^18^. In this study, we examine the relationship between the vaginal microbiota measured at two different time points and persistent hrHPV infection in a nested case control study of HIV-negative and HIV-positive Nigerian women.

## Results

### Study population

**Figure 1:**
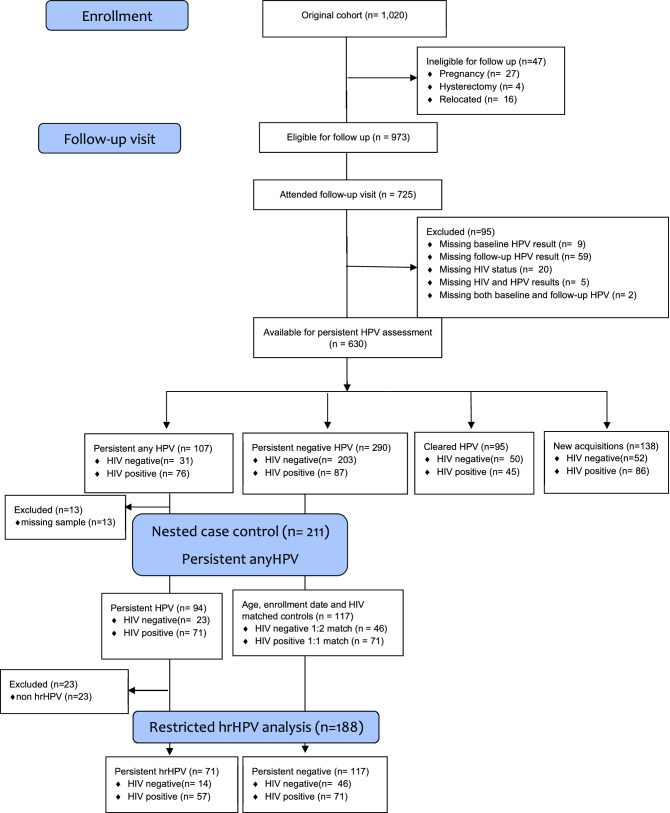
Participant flow chart and selection of participants in nested case control analysis.

From a prospective population—based cohort study of 1020 women, we selected 211 women (94 cases of persistently any HPV infected and 117 controls of persistently HPV negative women) for inclusion in this nested case control study using a 1:1 match for HIV positive women and a 1:2 match for HIV negative women. Details of participant selection are provided in Fig. 1.

Table 1 shows the baseline characteristics of the study population stratified by HIV status. Mean (SD) age of participants at enrolment was 38 (8) years while mean age at sexual debut was 20 (3) years. Most participants had more than six years of formal education (88%, 186/211) and were premenopausal (84%, 175/211). More than half of the participants were married (58%, 122/211) and douche regularly (60%, 126/211). The median interval (IQR) between baseline and follow-up visit was 6 (6–8) months.

**Table 1:** Baseline characteristics of study population by HIV status.

	All participants n = 211 (%)	HIV negative n = 69 (%)	HIV positive n = 142 (%)
	Mean, SD		
Age, years	37.8 (8.25)	37.6 (9.03)	38.0 (7.86)
Age at sexual debut, years	19.7 (3.47)	20.7 (3.99)	19.2 (3.08)
Lifetime number of sexual partners	3.94 (3.84)	3.01 (2.02)	4.40 (4.40)
Number of partners in preceding year	1.04 (0.60)	1.01 (0.44)	1.05 (0.67)
	n (%)		
**Socioeconomic status**			
Low	95 (45.0)	22 (31.9)	73 (51.4)
Middle	82 (38.9)	27 (39.1)	55 (38.7)
High	34 (16.1)	20 (29.0)	14 (9.86)
**Education, years**			
≤ 6	25 (11.9)	8 (11.6)	17 (12.0)
7 – 12	58 (27.5)	10 (14.5)	48 (33.8)
> 12	128 (60.7)	51 (73.9)	77 (54.2)
**Marital status**			
Unmarried	89 (42.2)	20 (29.0)	69 (48.6)
Married	122 (57.8)	49 (71.0)	73 (51.4)
**Menopause**			
No	175 (83.7)	55 (80.9)	120 (85.1)
Yes	34 (16.3)	13 (19.1)	21 (14.9)
**Time since last menstrual period, days**			
≤ 15	78 (49.7)	23 (46.9)	55 (50.9)
> 15	79 (50.3)	26 (53.1)	53 (49.1)
**Douche regularly**			
No	83 (39.7)	32 (46.4)	51 (36.4)
Yes	126 (60.3)	37 (53.6)	89 (63.6)
Douche material*			
Water	96 (76.2)	25 (67.6)	71 (79.8)
**Contraceptive use**			
None	86 (40.8)	39 (56.5)	47 (33.1)
Condoms	95 (45.0)	18 (26.1)	77 (54.2)
Oral Contraceptive	22 (10.4)	7 (10.1)	15 (10.6)
Hormonal injections	16 (7.58)	5 (7.3)	11 (7.75)
**Body mass index, kg/m**^**2**^** (N, %)**			
Normal weight, 18.5–24.9	65 (32.3)	14 (21.2)	51 (37.8)
Overweight, 25.0–29.9	83 (41.3)	27 (10.9)	56 (41.5)
Obese, ≥ 30.0	53 (26.4)	25 (37.9)	28 (20.7)
**Sexual activity within 24 h of sampling**			
Vaginal sex	22 (10.4)	11 (15.9)	11 (7.8)
Oral sex	3 (1.42)	2 (2.90)	1 (0.70)
Digital penetration	25 (11.9)	14 (20.3)	11 (7.8)
**Persistent any HPV**			
No	117 (55.4)	46 (66.7)	71 (50.0)
Yes	94 (44.6)	23 (33.3)	71 (50.0)
**Persistent hrHPV**			
Type-specific persistence	62 (29.4)	12 (17.4)	50 (35.2)
Group persistence	9 (4.3)	2 (2.9)	7 (4.9)

*Proportion of women who douche with water among women who regularly douche.

Of the 117 controls (women who were persistently negative for HPV), 39% (46/117) were HIV negative. Of the 94 cases (women who were persistently positive for any HPV), 24% (23/94) were HIV negative. Among women with persistent any HPV infection, 76% (71/94) had persistent hrHPV infections.

Among cases, the commonest hrHPV types detected were HPV 52 and HPV 35 at both baseline (27%, 25/94 and 23%, 22/94 respectively) and follow up visits (31%, 29/94 and 19%, 18/94 respectively). Details of the frequency distribution of other high risk HPV types identified in women with persistent any HPV infection at baseline and follow up stratified by HIV status are shown in Table 2. In women with persistent hrHPV infection, type specific persistence (same hrHPV type present at baseline and follow up) was commoner (87%, 62/71) than group persistence (13%, 9/71). The commonest hrHPV types in type specific persistence were HPV 52 (31%, 22/71) and HPV 35 (24%, 17/71). Further details of HPV prevalence, persistence and clearance of the parent cohort have been previously published^25,26^.

**Table 2:** Distribution of hrHPV types in 94 women with persistence of any HPV.

HPV type	Total n = 94 (%)	HIV negative n = 23 (%)	HIV positive n = 71 (%)
**Distribution of hrHPV types at baseline visit**			
16	6 (6.4)	0 (0.0)	6 (8.4)
18	9 (9.6)	2 (8.7)	7 (9.9)
31	9 (9.6)	2 (8.7)	7 (9.8)
33	9 (9.6)	2 (8.7)	7 (8.7)
35	22 (23.4)	3 (13.0)	19 (26.8)
39	3 (3.2)	0 (0.0)	3 (4.2)
45	4 (4.3)	1 (4.4)	3 (4.2)
51	7 (7.4)	1 (4.4)	6 (8.4)
52	25 (26.6)	6 (26.1)	19 (26.8)
56	5 (5.3)	2 (8.7)	3 (4.2)
58	5 (5.3)	2 (8.7)	3 (4.2)
59	5 (5.3)	0 (0.0)	5 (7.4)
**Distribution of hrHPV types at follow up visit**			
16	8 (8.5)	0 (0.0)	8 (11.3)
18	11 (11.7)	1 (4.4)	10 (14.1)
31	13 (13.8)	2 (8.7)	11 (15.5)
33	5 (5.3)	1 (4.4)	4 (5.6)
35	18 (19.2)	3 (13.0)	15 (21.1)
39	4 (4.3)	1 (4.4)	3 (4.2)
45	4 (4.3)	1 (4.4)	3 (4.2)
51	7 (7.5)	0 (0.0)	7 (9.9)
52	29 (30.9)	7 (30.4)	22 (31.0)
56	2 (2.1)	0 (0.0)	2 (2.8)
58	5 (5.3)	2 (8.7)	3 (4.2)
59	1 (1.1)	0 (0.0)	1 (1.4)

There were 422 visits made by the 211 participants selected. Approximately, 84% (353/422) of vaginal microbiota samples taken at these visits were available for 16S rRNA sequence analysis which yielded 12, 967, 629 high quality reads. The mean (SD) number of reads per sample was 36, 021 (14, 375), with a mean (SD) read length of 427 (6) base pairs.

### Vaginal microbiome composition in the study population

**Figure 2:**
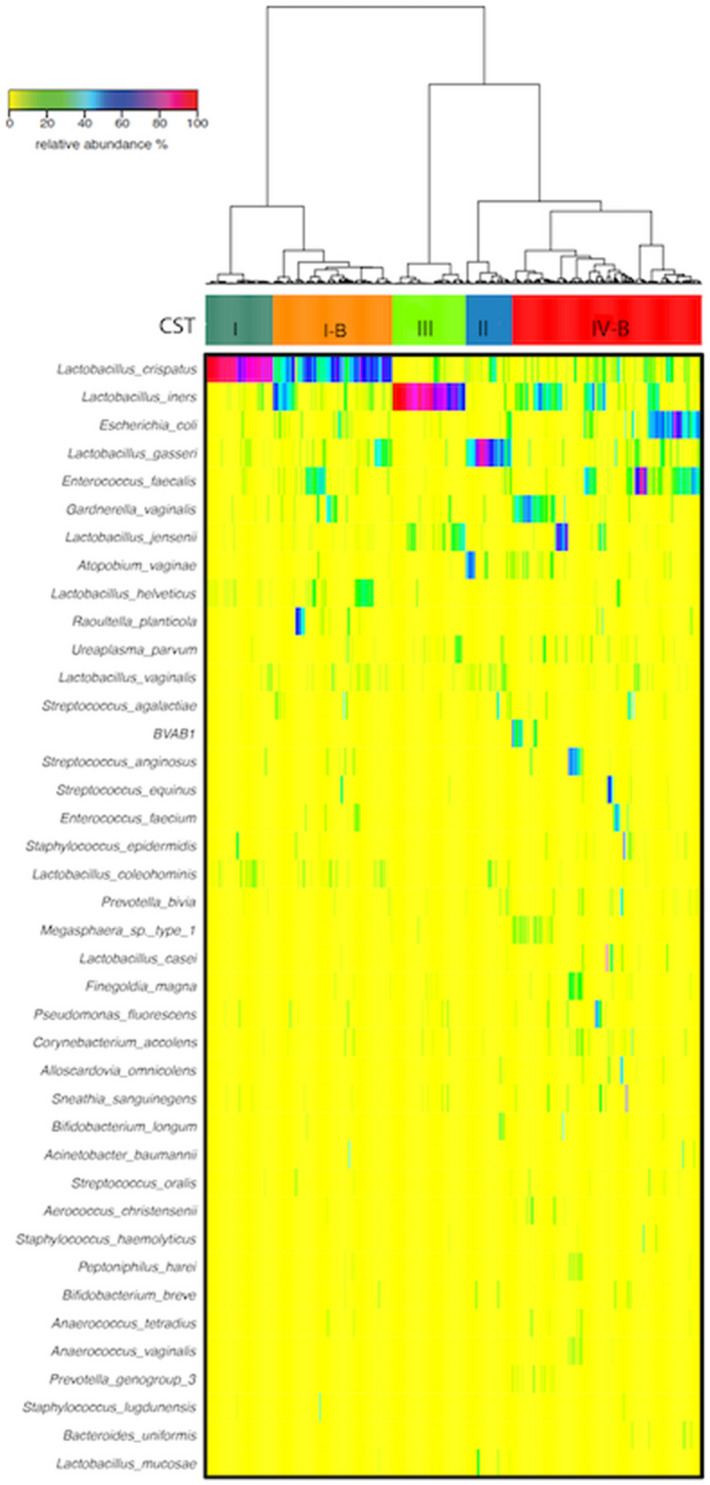
Heatmap of the 40 most abundant bacterial taxa in 211 Nigerian women. Hierarchical clustering identified five major clusters. Three of these clusters were dominated by one *Lactobacillus* species: *L.crispatus* (CST I), *L.gasseri* (CST II) and *L.iners* (CST III). CST I-B and CST IV-B were more diverse with CST I-B having modest proportions of *L.crispatus.* Ward linkage clustering was used to cluster samples based on their Jensen-Shannon distance calculated in the Vegan package in R^66^.

Hierarchical clustering analysis based on species composition and relative abundance identified five major clusters that showed similarities to previously described vaginal microbiome community state types (CST)^23^. Three of these CSTs were dominated by high proportions of *Lactobacillus* specie: *L. crispatus* in CST I, *L. gasseri* in CST II and *L. iners* in CST III (Fig. 2). CST I-B had moderately high proportions of *Lactobacillus* species, most commonly *L. crispatus* as well as low proportions of pathobionts such as *Enterococcus and Streptococcus,* which are not commonly seen in CST I. CST IV-B was characterized by greater taxonomic diversity with low proportions of *Lactobacillus*, pathobionts such as *Escherichia, Enterococcus and Streptococcus* as well as bacterial vaginosis associated taxa such as *Gardnerella, Atopobium, Megasphaera, Sneathia* and *Prevotella.*

**Figure 3:**
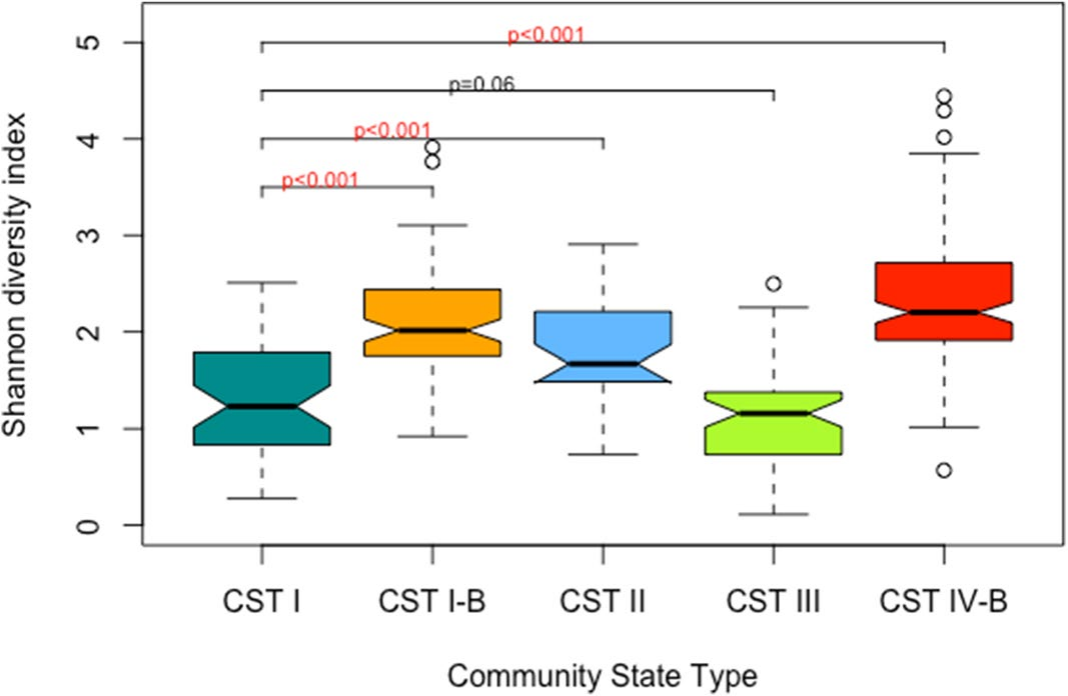
Shannon diversity indices as a measure of alpha diversity by community state types. Compared to CST I, alpha diversity is significantly higher in CST II, CST I-B and CST IV-B.

**Figure 4:**
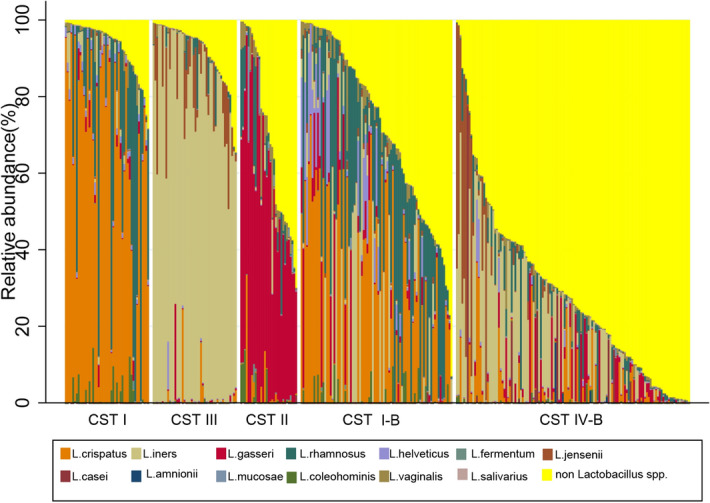
Relative abundance of *Lactobacillus* species by Community State Assignments. *Lactobacillus* species were present in all communities. CST I-B had modest proportions of *L.crispatus* as well as other *Lactobacillus* species, most commonly *L.rhamnosus.* CST IV-B was the most diverse community with higher proportions of non-*Lactobacillus* species.

Alpha diversity was significantly lower in CST I compared to CST II (*p* < 0.001), CST I-B (*p* < 0.001) and CST IV-B (*p* < 0.001). There were no significant differences in alpha diversity between CST I and CST III (p = 0.06) (Fig. 3). *Lactobacillus* species were present in all CSTs (Fig. 4). The median (IQR) relative abundance of *Lactobacillus* species was 97% (90–98%) in CST I; 75% (50–93%) in CST II; 95% (87–98%) in CST III; 77% (57–95%) in CST I-B and 27% (11–42%) in CST IV-B.

In HIV negative women, the point prevalence of CST IV-B was 38% (26/69) at the baseline visit and 30% (21/69) at the follow up visit (Table 3). The difference in proportions (0.12; 95% CI − 0.06–0.30; *p* = 0.20) was not statistically significant. In HIV positive women, the point prevalence of CST IV-B was 27% (38/142) at the baseline visit and 35% (50/142) at the follow up visit. This CST distribution at the baseline and follow up visit was not statistically significant (difference in proportions = 0.06, 95% CI = − 0.10–0.14, *p* = 0.75). Of the *Lactobacillus* species dominant CSTs, CST III was the most prevalent at both baseline (15%, 10/69) and follow up (30%, 21/69) visits in HIV negative women. In HIV positive women, the most prevalent *Lactobacillus* species dominant CST was CST III at the baseline visit (9%, 13/142) and CST II at the follow up visit (15%, 21/142) (Table 3).

**Table 3:** CST characterization in the study population by HIV and HPV status in women persistently negative for HPV (HPV–), persistently positive for hrHPV (hrHPV + +) and persistently positive for non hrHPV (non hrHPV + +).

Vaginal community at baseline								
	HIV negative women				HIV positive women			
	N = 69 (%)	HPV−− n = 46 (%)	hrHPV++ n = 14 (%)	Non hrHPV++ n = 9 (%)	N = 142 (%)	HPV−− n = 71 (%)	hrHPV++ n = 57 (%)	Non hrHPV++ n = 14 (%)
CST I	6 (9)	2 (4)	1 (7)	3 (33)	11 (8)	4 (6)	6 (11)	1 (7)
CST II	7 (10)	5 (11)	0 (0)	2 (22)	3 (2)	1 (1)	2 (4)	0 (0)
CST III	10 (15)	7 (15)	2 (14)	1 (11)	13 (9)	7 (10)	5 (9)	1 (7)
CST I-B	4 (6)	4 (9)	0 (0)	0 (0)	37 (26)	19 (27)	16 (28)	2 (14)
CST IV-B	26 (38)	17 (37)	9 (64)	0 (0)	38 (27)	17 (24)	17 (30)	4 (29)
Missing CST	16 (23)	11 (24)	2 (14)	3 (33)	40 (28)	23 (32)	11 (19)	6 (43)

Vaginal community at follow-up								
	HIV negative women				HIV positive women			
	N = 69 (%)	HPV−− n = 46 (%)	hrHPV++ n = 14 (%)	Non hrHPV++ n = 9 (%)	N = 142 (%)	HPV−− n = 71 (%)	hrHPV++ n = 57 (%)	Non hrHPV++ n = 14 (%)
CST I	11 (16)	7 (15)	1 (7)	3 (33)	20 (14)	10 (14)	9 (16)	1 (7)
CST II	2 (3)	1 (2)	1 (7)	0 (0)	21 (15)	7 (10)	8 (14)	6 (43)
CST III	21 (30)	15 (33)	5 (36)	1 (11)	8 (6)	4 (6)	4 (7)	0 (0)
CST I-B	2 (3)	2 (4)	0 (0)	0 (0)	42 (32)	25 (35)	16 (28)	1 (7)
CST IV-B	21 (30)	13 (28)	5 (36)	3 (33)	50 (35)	25 (35)	20 (35)	5 (36)
Missing CST	12 (17)	8 (17)	2 (14)	2 (22)	1 (0)	0 (0)	0 (0)	1 (7)

*N* All participants included in analysis.

*HPV−−* Negative for HPV at both baseline and follow up visits.

*hrHPV++* Positive for hrHPV at both baseline and follow up visits.

*Non hrHPV++* Persistently positive for non hrHPV at baseline and follow up visits. This group includes women positive for low risk HPV at baseline and positive for low risk HPV at follow up; positive for low risk HPV at baseline and positive for hrHPV at follow up; positive for hrHPV at baseline and positive for low risk HPV at follow-up.

**Figure 5:**
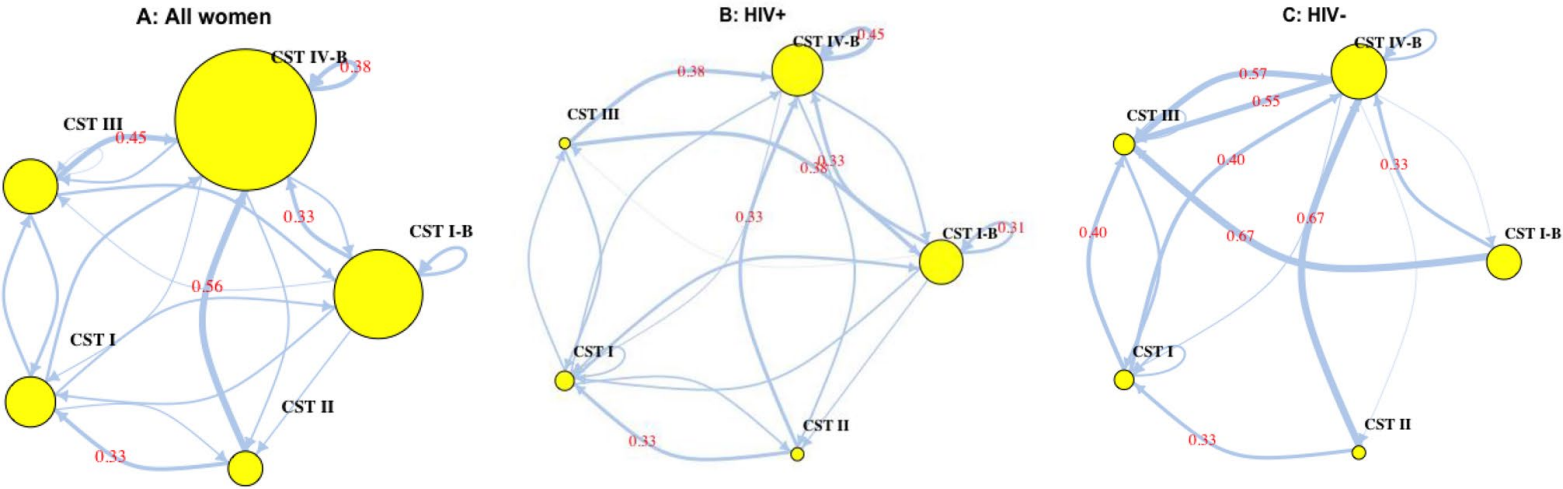
Markov chain transition probabilities over two time periods, approximately six months apart by HIV status. Arrow weights are proportional to the maximum likelihood estimate of the transition probability between CSTs at the baseline and follow up visits. Node sizes scale with the number of women in each CST. Numeric transition probabilities are shown on arrows for transition probabilities > 0.30. Markov chain schematic transitions were modelled using the markovchain package in R^67^.

Markov chain transition probabilities indicated substantial variability in inter-CST transitions over the two-time points (baseline and follow-up visits) (Fig. 5). Patterns of transition probabilities were comparable among HIV positive and HIV negative women. In both HIV negative and HIV positive women, the highest inter-state transition probabilities were to the more diverse community (CST IV-B). While the highest self-transition probability, the probability that a CST would be the same at both baseline and follow up, was observed for CST IV-B in both HIV negative and HIV positive women, indicating that CST IV-B was the most stable CST among women in this study.

In HIV negative women, transition probabilities from other CSTs to CST IV-B were 0.40 (CST I), 0.67 (CST II), 0.57 (CST III), and 0.33 (CST I-B). There were no transitions from a *Lactobacillus* dominant CST (CST I, CST II, CST III) to CST I-B, while CST II was the least connected CST. Transitions from CST IV-B were most commonly to CST III (transition probability = 0.55). In HIV positive women, transition probabilities from other CSTs to CST IV-B were 0.18 (CST I), 0.33 (CST II), 0.38 (CST III) and 0.33 (CST I-B). There were no transitions from CST II to CST I-B.

### Association between demographic, lifestyle and clinical characteristics and the vaginal microbiome

We evaluated potential demographic and behavioural correlates for a *Lactobacillus* species dominant microbiota and a *Lactobacillus crispatus* dominant microbiota, treating the vaginal microbiota as a time varying response variable with measurements at two time points – baseline and follow up visits (Table 4). In univariate analyses, being HIV-positive was significantly associated with reduced odds of having a *Lactobacillus* species dominant microbiome (OR: 0.53, 95% CI 0.34–0.84, *p* = 0.007) or a *L.crispatus* dominant microbiome (OR: 0.33, 95% CI 0.15–0.72, *p* = 0.006). Adjustments for age in multivariable models did not significantly change this relationship. Other than HIV status, there were no other associations between demographic, lifestyle, or clinical characteristics and the vaginal microbiota.

**Table 4:** Association between potential risk factors and vaginal microbiome.

Characteristic	Total^‡^	Lactobacillus dominant microbiota*			*L. crispatus* dominant microbiota**		
	n^†^	n^†^ (%)	OR (95% CI)	*p* value	n^†^ (%)	OR (95% CI)	*p* value
Age, years	–	–	1.00 (0.97–1.03)	0.92	–	1.01 (0.97–1.06)	0.56
**Socioeconomic status**							
Low	149	75 (50)	1.00		10 (7)	1.00	
Middle	145	70 (48)	0.92 (0.57–1.48)	0.73	10 (7)	1.03 (0.42–2.50)	0.95
High	59	29 (39	0.95 (0.52–1.74)	0.88	5 (8)	1.29 (0.43–3.81)	0.65
**Education, years**							
≤ 6	39	15 (38)	1.00		2 (5)		
7–12	100	47 (47)	1.42 (0.68–2.95)	0.35	3 (3)	0.57 (0.09–3.64)	0.55
> 12	214	112 (52)	1.76 (0.89–3.46)	0.10	20 (9)	1.91 (0.42–8.71)	0.41
**Menopausal**							
No	294	141 (48)	1.00		17 (5)	1.00	
Yes	48	26 (54))	1.28 (0.65–2.53)	0.47	5 (10)	1.89 (0.68–5.23)	0.22
**Time since last menstrual period, days**							
≤ 15	119	53 (45)	1.00		6 (5)	1.00	
> 15	125	58 (46)	1.08 (0.64–1.83)	0.78	8 (6)	1.29 (0.43–3.87)	0.65
**Douche regularly**							
No	141	64 (45)	1.00		10 (7)	1.00	
Yes	156	79 (51)	1.23 (0.78–1.96)	0.37	10 (6)	0.90 (0.36–2.21)	0.81
**Body Mass Index, kg/m**^**2**^							
Normal weight, 18.5–24.9	110	52 (47)	1.00		5 (5)	1.00	
Overweight, 25.0–29.9	144	75 (52)	1.21 (0.73–2.00)	0.45	10 (7)	1.57 (0.54–4.59)	0.41
Obese, ≥ 30.0	86	39 (45)	0.93 (0.51–1.67)	0.80	8 (9)	2.15 (0.70–6.64)	0.18
**HIV status**							
Negative	110	66 (60)	1.00		14 (13)	1.00	
Positive	243	108 (44)	0.53 (0.34–0.84)	0.007	11 (5)	0.33 (0.15–0.72)	0.006
**Sex within 24 h**							
No	305	150 (49)	1.00		23 (8))	1.00	
Yes	46	23 (50)	1.08 (0.61–1.91)	0.80	1 (2)	0.69 (0.20–2.40)	0.56
Total sex partners in lifetime	–	–	0.98 (0.92–1.04)	0.45	–	0.97 (0.89–1.07)	0.61
Total sex partners in 1 year	–	–	1.04 (0.71–1.53)	0.83	–	0.71 (0.34–1.51)	0.38
**Condom use**							
No	192	100 (52)	1.00		16 (8)	1.00	
Yes	74	74 (46)	0.78 (0.51–1.20)	0.26	9 (6)	0.65 (0.29–1.48)	0.31
**Oral contraceptive use**							
No	315	156 (50)	1.00		24 (8)	1.00	
Yes	38	18 (47)	0.92 (0.44–1.92)	0.82	1 (3)	0.33 (0.04–2.55)	0.29

*Relative abundance of *Lactobacillus* species > 70%.

**Relative abundance of *L. crispatus* > 70%.

^†^Frequency of exposure at the two visits combined. The outcomes and some variables (oral contraceptive use, condom use, total sex partners in 1 year, total sex partners in lifetime, sex within 24 h, BMI, douche regularly, time since last menstrual period, menopausal state) were modelled as time varying covariates. Correlation within person measurements were accounted for in GEE models.

Given the variability between participants that clustered in CST groups, we used a marker of community functionality—hence analysis by relative abundance of *Lactobacillus.* 70% is used at cut off because of the distribution of the relative abundance of *Lactobacillus* in the different CST groups.

^‡^59 participants were missing vaginal microbiota at baseline, and 13 participants were missing vaginal microbiota at follow up.

### Association between persistent hrHPV infection and vaginal microbiota

A priori, we considered the possibility of HIV modifying the relationship between the vaginal microbiota and persistent hrHPV based on results from previous studies and biological plausibility^18, 27^. In effect modification analysis, the odds ratio of having persistent hrHPV for women with a *Lactobacillus* dominant microbiota (> 70%) versus a *Lactobacillus* depleted microbiota was multiplied by a factor of 3.39 (95% CI 1.18–9.70, *p* = 0.02) for HIV-positive women as compared to HIV-negative women. Therefore, we present effect estimates by HIV strata.

Among HIV negative women, the odds of persistent hrHPV infection were significantly lower in women whose microbiota were dominated by *Lactobacillus* species (OR: 0.35, 95% CI: 0.14–0.89, *p* = 0.03) than women with *Lactobacillus* depleted microbiota. In HIV positive women, there was no significant association between persistent hrHPV infection and the vaginal microbiota in the *Lactobacillus* dominant model (OR: 1.25, 95% CI: 0.73–2.14, *p* = 0.41) (Table 5). There were also no significant associations between the vaginal microbiota and persistent hrHPV in the other models (*L.crispatus* dominant [> 70%] model and Community State Assignment model) considered for both HIV-negative and HIV-positive women.

**Table 5:** Association between persistent hrHPV infection and vaginal microbiota.

	Persistent hrHPV			
	HIV negative		HIV positive	
	OR (95% CI)	*p*	OR (95% CI)	*p*
Model 1*				
*Lactobacillus* dominant microbiota				
< 70%	1.00		1.00	
≥ 70%	0.35 (0.14–0.89)	0.03	1.25 (0.73–2.14)	0.41
Model 2*				
L.crispatus dominant microbiota				
< 70%	1.00		1.00	
≥ 70%	0.22 (0.03–1.43)	0.11	1.20 (0.82–1.76)	0.34
Model 3*				
Community State Assignment				
CST IV-B	1.00		1.00	
CST I-B	–		0.95 (0.50–1.82)	0.88
CST III	0.67 (0.28–1.61)	0.38	1.03 (0.39–2.70)	0.95
CST II	0.28 (0.04–2.02)	0.21	1.00 (0.40–2.55)	0.99
CST I	0.29 (0.06–1.46)	0.13	1.30 (0.40–2.54)	0.47

*Each model is adjusted for age.

In sensitivity analyses varying cut points to dichotomize relative abundance of *Lactobacillus* species, with values ranging from 50 to 80% in increments of 5%, the association between *Lactobacillus* dominated microbiota and persistent hrHPV in HIV negative women was insensitive to cut off points between 50 and 80% indicating that results were robust within this range of cut points (Supplementary Table 1). Analysis of characteristics of participants missing microbiome data indicated that missingness at baseline was associated with socioeconomic status; while missingness at follow up was associated with HIV status (Supplementary Table 2). These variables (socioeconomic status, study visit and HIV status) were used as auxiliary variables during multiple imputation for sensitivity analysis. Effect estimates from the imputed data were similar to effect estimates obtained from complete case analysis (Supplementary Table 3).

**Figure 6:**
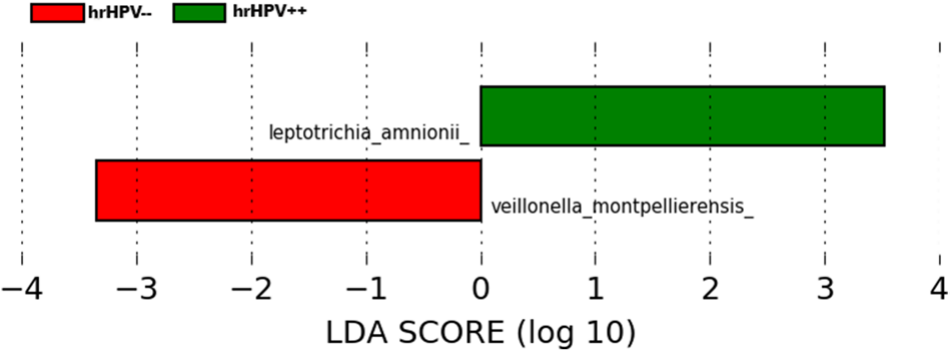
Identified phylotype biomarkers of persistent hrHPV infection among HIV negative women. The phylotype biomarkers are identified as being significantly abundant at alpha value < 0.05. The analysis was run and visualized with the LEfSe program using the Galaxy interface^68^. The Linear Discriminant Analysis (LDA) score is indicated at the bottom. The greater the LDA score, the more significant the phylotype biomarker is.

We used LEfSe modelling to identify specific bacterial taxa that may be differentially abundant in HIV negative women with and without persistent hrHPV; and HIV positive women with and without persistent hrHPV. We included HIV positive women in LEfSe analysis as the broad categories in the regression models may mask differences in non-dominant bacterial taxa. We found that in HIV-negative women, women with persistent hrHPV infection had significant over-representation of *Leptotrichia* species in their vaginal microbiota compared to HIV-negative women without persistent hrHPV infection (Fig. 6) Conversely, there was significant overrepresentation of *Veillonella* species in HIV-negative women who were persistently hrHPV negative compared to HIV negative women with persistent hrHPV infection (Fig. 6) . In HIV-positive women, we did not identify any discriminative features between the vaginal microbiota of women with persistent hrHPV infection and women who were persistently HPV negative.

## Discussion

In this nested case control study of the vaginal microbiota and persistent hrHPV infection in HIV-negative and HIV-positive women, we found that a high relative abundance of *Lactobacillus* species was associated with reduced odds of persistent hrHPV infection in HIV-negative women but not in HIV-positive women. These results extend findings from our previous study, in which we showed a significant association between a low relative abundance of *Lactobacillus* species and increased odds of prevalent hrHPV, a more proximal causal intermediate in the relationship between hrHPV and cervical cancer^18^. Consistent with our previous report on prevalent hrHPV and other studies, we also identified *Leptotrichia* species to be over-represented in HIV-negative women with persistent hrHPV^18,21^.

While recent longitudinal studies with repeated vaginal microbiota assessments that use next generation sequencing techniques have shown an association between higher vaginal microbial diversity and prevalent hrHPV or persistent any HPV, our study is the first to show this for persistent hrHPV^19,24^. In one study conducted among Italian women, Di Paola et. al. provide evidence to show that a high diversity vaginal microbiota, CST IV, at baseline was predictive of future odds of persistent hrHPV, despite not accounting for the temporal dynamics of the vaginal microbiota^21^. Several other cross-sectional studies have showed that decreased relative abundance of *Lactobacillus* species is associated with precancerous cervical lesions and cervical cancer^28–30^. Collectively, these results suggest that the vaginal microbiome may play a role in cervical cancer pathogenesis.

It is plausible that the vaginal microbiome modulates risk of HPV acquisition^31^, although there is limited mechanistic evidence for this relationship. It is also possible, though less probable, that the presence of HPV interacts with the host immune system through complex mechanisms that lead to the presence of immunosuppressive cytokines that favour different vaginal communities^17,32,33^. Alternatively, sexual activity, a common cofactor for both vaginal microbiota and HPV infection may explain the associations observed.

Consistent with previous studies on the temporal dynamics of the vaginal microbiota, we found that CST IV-B was the most stable CST and when transitions did occur, they were most commonly to the *L.iners* dominated CST III^23^. These findings of the stability of CST IV-B may be considered to be qualitatively similar to a longitudinal study among 80 African women which showed that high diversity associated bacteria such as *Prevotella bivia* and *Gadnerella vaginalis* were consistently present in 91% and 58% of healthy women with a Nugent score of 0–3 respectively, while *L. crispatus* was consistently present in only 47%^34^. These findings may reflect a lack of optimal protection against the growth of strict anaerobes by *L. iners or* the ability of *L.iners* to thrive in a range of metabolic stress related conditions that other *Lactobacillus* species cannot survive in^35^. Future studies on the functional metabolomics profile associated with *L. iners* may help to understand the role of this specie in the maintenance and potential restoration of a healthy vaginal microbiota.

We found that HIV-positive women were less likely to have a high relative abundance *of Lactobacillus* species or *L. crispatus* compared to HIV-negative women. Microbial diversity has been reported to be an important risk factor for HIV acquisition and viral shedding^20,36,37^. Recent reports, in which HIV susceptible CD4 + cells are increased in the genital tract in women with high diversity microbial communities, provide further mechanistic evidence for the link between increased risk of HIV acquisition and viral shedding^15,16^. In addition, it is possible that the lower relative abundance of *Lactobacillus* species among HIV positive women in our study was due to immunosuppression resulting in subsequent colonization of genital tract by diverse bacteria.

Similar to previous studies, we did not find any significant association between vaginal douching and the vaginal microbiota^20,34,38,39^. The influence of hygienic practices such as vaginal douching on the microbiota may be mediated by factors such as the type of materials used for douching and timing in the menstrual cycle^40,41^. In a meta-analysis of intra-vaginal practices and bacterial vaginosis, which included 13 prospective studies in Sub Saharan Africa, vaginal douching with soap, but not other materials, was associated with bacterial vaginosis^41^. In our study population, majority of women who reported douching regularly, used water to douche, providing some explanation for our findings.

In this analysis, we did not find any association between lifetime number of sexual partners, number of partners in past year, recent sexual activity and the vaginal microbiota. Previous studies have reported reduced *Lactobacillus* species in women with recent sexual activity^23,34^. Our inability to detect these relationships may be due to the small sample size of women who reported recent sexual activity in our study.

Among HIV negative women, results from LEfSe analysis indicated that while *Leptotrichia* species was differentially more abundant in persistent hrHPV infected women, *Veillonella* species was differentially more abundant in hrHPV negative women. The association between *Letptotrichia* species and hrHPV infection has been reported in previous studies^18,21^. Less frequently reported is the observation of a higher abundance of *Veillonella* in hrHPV negative women. *Veillonella* are anaerobic, gram negative cocci commonly found in the oral cavity, gut and vaginal tract that play diverse roles in different niches^42^. The pathogenic role of *Veillonella* is uncertain and they are currently considered to be of low virulence^43,44^. While *Veillonella* species have been associated with more diverse microbial communities, including the formation of biofilms leading to dental plaques in the oral cavity and serious infections such as endocarditis^45^, they have also been found to be more abundant in HPV negative women compared to HPV positive women^13,46^, as well as in the oral microbiota of HPV negative infants compared to the oral microbiota of HPV positive infants^46^. Paradoxically, while they may be less common in HPV positive vaginal microbiota, they are increasingly more abundant in distal end points in the natural history of cervical cancer pathogenesis, such as in precancerous lesions^13^, Bogert et al*.* observed that the cytokine response profile of *Veillonella* species within the gut microbiota is dependent on the combinations of the bacteria species present^47^. These findings taken together with the paradoxical observations, may highlight the interplay between the integrated responses to multiple representative species within a microbial environment in influencing disease risk and warrant a deeper functional characterization of the vaginal microbiota.

The lack of association between *Lactobacillus* as a genus and hrHPV negative status in LEfSe analysis is noteworthy, given the strong associations between *Lactobacillus* species and hrHPV negative status that has been reported in previous studies. However, in this population, it must be noted that the commonest and the most stable microbial communities was the diverse CST IV-B community. This provides credence to current understanding that even in diverse communities, the important catabolic function, of lactic acid production, is conserved among communities despite differences in species composition.

Definitions of HPV persistence vary considerably across studies^48^. The commonest definitions include HPV positivity measured at two or more time points with an interval of six or more months^48^. At one extreme of this definition is the requirement for the same HPV genotype to be present at both time points (genotype-specific persistence), and the other extreme is the presence of any HPV at both time points (group persistence). Conceivably, HPV genotype-specific persistence is better suited for identifying women at increased risk of cervical cancer and its precursor lesions, as group persistence could be the result of one HPV type clearing and another being acquired. However, results from a population-based cohort study in Denmark have shown that women with hrHPV group persistence have increased odds of high-grade precancerous lesions that are almost 200-fold higher than observed in women persistently negative for HPV^49^. Furthermore, in studies investigating the use of group persistence versus genotype specific persistence to inform referral practices, the use of genotype specific persistence did not improve risk stratification over the use of group persistence in women referred for colposcopy indicating that group persistence is a good proxy for measuring type-specific persistence and risk of high grade precancerous lesions^50,51^.

While our study is the single largest nested case control study of persistent hrHPV and the vaginal microbiota among HIV-negative and HIV-positive women in Africa to date, it has some limitations. As with all case control studies, there is potential for selection bias when the selected cases and controls are systematically different from the target population especially when the selection criteria are associated with both the outcome and the exposure of interest^52^. In this study, the risk of selection bias is minimized for two reasons. First the parent cohort is a longitudinal population-based cohort; cases and controls selected from this cohort are representative of the source population with regards to exposure—outcome relationships. However, the prevalence of the exposure and outcome may vary substantially between the selected study population and the source population. Therefore, we limit our inferences to associations between exposure and outcome and make no inferences about the generalizability of the prevalence estimates of hrHPV or vaginal microbiota observed in this population. Secondly, while our selection criterion was associated with the outcome (all cases were selected), it was not associated with the exposure of interest. This reduces the risk of bias due to selection.

We included microbiome data from only two time points and did not perform qPCR to determine concentration. The human vaginal microbiota composition can be highly variable over short periods of time due to several factors, therefore sampling over shorter time intervals for extended periods are required. However, we did not find significant transitions of CST-IV to other CSTs, suggesting that additional sampling among women with CST IV may not have changed our results. There was some degree of missingness for vaginal microbiota data which could be a source of bias if differential. However, results from sensitivity analysis where missing vaginal microbiota was imputed based on visit (baseline or follow up), socioeconomic status and HIV status was not markedly different from the results based on complete non-missing data. We assessed HIV status at enrolment into the study but did not repeat HIV testing at follow up. It is possible that some women classified as HIV negative at the beginning of the study seroconverted during follow up, resulting in misclassification. We did not account for the CD4 + counts, viral loads, ART use or duration of infection in the HIV positive women included in this study and these may have affected our findings for HIV positive women.

In summary, we found that HIV negative women with a high relative abundance of *Lactobacillus* species in the vaginal microbiota had lower odds of persistent hrHPV compared to HIV negative women with lower relative abundance of *Lactobacillus* species. There was also an inverse relationship between the relative abundance of *Lactobacillus* species and HIV infection, such that women who were HIV-positive had lower odds of a *Lactobacillus* specie dominant vaginal microbiota. Further work is required to understand the functional profile of the vaginal microbial communities and how this interacts with host factors to modulate risk of hrHPV persistence in the pathogenesis of cervical cancer.

## Methods

### Study population

We enrolled 1,020 women from our cervical cancer screening studies into a prospective study of host and viral factors associated with persistent hrHPV infection in Abuja, Nigeria between 2012 and 2013^53,54^. We included non-pregnant women who were at least 18 years old, had engaged in vaginal sexual intercourse and had no previous history of hysterectomy or cervical cancer. Trained nurses collected information on sociodemographic characteristics, self-reported HIV status, lifestyle factors, sexual behaviour and reproductive history from all participants. Participants were scheduled for a follow-up visit six months after the baseline visit. At both baseline and follow-up visits, nurses performed gynaecological examinations and collected biological specimens.

For this analysis, we used a nested case control design restricted to 211 women selected as cases and controls. Details of patient selection are provided in Fig. 1. Of the 1020 women in the parent cohort, 23 HIV negative women and 71 HIV positive women were persistently positive for any HPV. All of these 94 women were selected as cases in this nested study. For each case we identified either one (for HIV positive cases) or two (for HIV negative cases) controls from the cohort, matched by age (± 5 years) and study enrolment date (± 5 days) resulting in 117 controls in total. These 211 women provided data at two clinic visits resulting in 422 visits.

### Sample collection and processing

We collected mid-vaginal swabs from all participants using the Elution Swab system (Copan, Italy) which was inserted in one ml Amies’ Transport media (Copan, Italy) and stored at − 80 °C till further processing. Whole genomic bacterial DNA was extracted from the swabs using the MoBio Powersoil kit, as previously described^18^.

### Bacterial 16S rRNA gene sequencing and analysis

A dual barcode system with fusion primers 338F and 806R was used to amplify the V3–V4 hypervariable regions of the 16S rRNA gene as previously described^55,56^. Purified amplicons were sequenced on an Illumina Miseq instrument (Illumina, San Diego, CA) using a 300 bp paired end protocol at the Institute for Genome Sciences, University of Maryland School of Medicine. Raw reads were pre-processed to remove the first three and last three bases if their Phred score was lower than 3. Read ends were trimmed if the average Phred quality score of four consecutive bases was below 15 and only retained if their length was at least 75% of their original length after trimming. QIIME (v1.8.0)^57^ was used to perform quality control of the sequence reads. Fast Length Adjustment of Short reads (FLASH)^58^ was used to assemble paired reads, with an overlap of ~ 90 bp on average. De-multiplexing by binning sequences with the same barcode was performed in QIIME (v1.8.0). Chimera detection was done using de novo and referenced-based methods in UCHIME (v5.1) with the Greengenes database of 16S rRNA gene sequences (August 2013) as a reference^59,60^. The processed 16S rRNA gene amplicon sequences were assigned to genera and species, using PECAN that uses fifth-order Markov Chain model for precise species-level assignments and a pre-compiled database that contains all known microbes in the vaginal microbiota. Raw, demultiplexed, sequence files are available at the NBCI Sequence Read Archive (https://www.ncbi.nlm.nih.gov/sra) with accession code PRJNA668516. Hierarchical clustering analysis based on Jensen Shannon distances using ward linkage was used to cluster samples into Community State Types (CSTs) using the vegan package in R^23^.

### HPV detection

We extracted DNA from ectocervical cells and used the SPF_10_LiPA_25_ system for HPV DNA detection^61^. Briefly, we performed polymerase chain reaction on DNA isolates using biotin labeled SPF_10_ primers which generated small amplicons of 65 bp. The amplified PCR products were tested using DNA Enzyme Immunoassay (DEIA), a probe hybridization assay containing a cocktail of conservative probes that allows the recognition of at least 65 HPV genotypes. The amplimers of the DEIA HPV DNA positive samples were subsequently analyzed by the reverse hybridization line probe assay, LiPA_25_, Version 1 (Laboratory Biomedical Products, Rijswijk, The Netherlands) which can identify 25 HPV types, 12 of which are high-risk: 16, 18, 31, 33, 35, 39, 45, 51, 52, 56, 58, and 59. In this analysis, we use group persistence as the definition of persistent hrHPV, that is, the presence of at least one hrHPV type at baseline and one hrHPV type at the follow up visit as opposed to type specific persistence, which is the presence of identical hrHPV types at the two visits.

### Covariates

We collected information on potential confounders such as menopausal state, time since last menstrual period, douching practice, total number of sexual partners in past year, body mass index (BMI), vaginal pH, sexual activity within 24 h of sampling, use of condoms and hormonal contraceptive use. Vaginal pH was assessed as an ordinal variable with three categories: < 4.5, 4.5–5.5 and > 5.5. These covariates were assessed at the baseline visit and updated at the follow up visit. They were included in the models as time varying exposures. The vaginal microbiota was also modelled as a time varying exposure. In addition to CST based models, we conducted analysis based on the relative abundance of *Lactobacillus* (at the genus level) and *L. crispatus.* We specifically investigated *L.crispatus* at the species level because, of all the species of the *Lactobacillus* genus, commonly identified in the vaginal microbiota, communities with high relative abundance of *L.crispatus* have the lowest vaginal pH values, a marker for important vaginal defence mechanisms^20,33,62^.

### Statistical analysis

We compared the characteristics of participants at baseline by HIV status. We used generalized estimating equation (GEE) logistic regression models with robust variance estimation and the cluster option, which accounts for repeated measures, to investigate the relationship between potential risk factors and the vaginal microbiota; and the association between the vaginal microbiota and persistent hrHPV infection. Variables considered for inclusion in multivariable regression models were selected using a two-step approach. First, sociodemographic characteristics and potential risk factors associated with persistent HPV infection at a nominal *p* < 0.10 in the parent cohort of this study were identified from previously published results^25^. The variables identified from this step were BMI, marital status, education, socioeconomic status, age at sexual initiation, total lifetime sex partners, vaginal douching, vaginal pH and HIV status. Next, we examined potential risk factors and its association with vaginal microbiota in this cohort of participants. Variables that were significantly associated with the vaginal microbiota at *p* < 0.10 were identified. Finally, variables that were associated with both persistent HPV infection and the vaginal microbiota were selected for inclusion in multivariable models.

In modelling the vaginal microbiota, we considered three approaches: dichotomising based on *L. crispatus* relative abundance (using a cut off of 70%), dichotomising based on all *Lactobacillus* species relative abundance (using a cut off of 70%) and using CST assignments. To select the cut-off point, we used the mean of the relative abundance of *L. crispatus* in CST I because *L. crispatus* is often considered a marker of a healthy microbiome^19,63^. We investigated the sensitivity of results to various cut points by varying the cut points in increments of 5% from 50 to 80%. The upper and lower bounds for these sensitivity analyses were selected to ensure that we reached a point where further dichotomization would become uninformative.

We used Markov chain models generated by inferring transition probabilities from our data to evaluate vaginal community dynamics over the two time periods, the baseline visit and the follow up visit.

We used Linear Discriminant Analysis (LDA) effect size (LEfSe) algorithm to identify bacterial taxa that were differentially present in women with persistent hrHPV infections^64^.

Of the 422 clinical visits included in this study, results for 69 vaginal microbiota samples were missing. We performed univariate analysis for missingness at baseline and at the follow up visit to determine whether missingness was associated with important covariates such as HIV status, persistent hrHPV, age, socioeconomic status, education, menopausal state, time since last menstrual period, douching practice, BMI, sex within the last 24 h prior to sample collection and total number of sexual partners in the past one year. Furthermore, to minimize the potential for selection bias, we imputed missing microbiota data using a data augmentation algorithm in a Markov Chain Monte Carlo (MCMC) procedure creating 10 datasets in 1000 iterations. We included three variables as auxiliary predictor variables (socioeconomic status, study visit and HIV status) identified from the univariate analysis of missingness. Next, we implemented GEE logistic regression models in the imputed dataset and compared results obtained from the imputed data with results obtained from complete case analysis.

Based on previous studies that have provided evidence of increased microbial diversity and a higher risk of persistent hrHPV infections among HIV positive women compared to HIV negative women^25,27,65^, we considered the possibility of HIV modifying the relationship between the vaginal microbiota and persistent hrHPV infection, a priori. We tested for this statistically by fitting an interaction term in the GEE logistic regression model and comparing the fitted model to a model without the interaction term using likelihood ratio tests. The interaction term was statistically significant (*p* = 0.02). Therefore, we present results by HIV strata.

All analyses were conducted using Stata/SE 15 (StataCorp, College Station, Texas) and R version 3.4.0 (R Core Team, Vienna, Australia).

### Ethics

All methods were carried out in accordance with relevant guidelines and regulations. We obtained ethical approval to conduct this study from the National Health Research Ethics Committee of Nigeria and the University of Maryland School of Medicine Institutional Review Board. All participants provided written informed consent prior to enrolment.

## Supplementary information

Supplementary Information
