# Supplementary material for: Vaginal microbiota diversity and paucity of Lactobacillus species are associated with persistent hrHPV infection in HIV negative but not in HIV positive women
Source: Sci Rep. 2020 Nov 5;10:19095. doi: 10.1038/s41598-020-76003-7 (PMC7644686; doi:10.1038/s41598-020-76003-7)
Supplement: Supplementary file 1 — Supplementary Information [file 41598_2020_76003_MOESM1_ESM.docx]

**Vaginal microbiota diversity and paucity of *Lactobacillus* species are associated with persistent hrHPV infection in HIV negative but not in HIV positive women**

Eileen O. Dareng^1,2^, Bing Ma^3^. Sally N. Adebamowo^4,5^, Ayotunde Famooto^2^, Jacques Ravel^3^, Paul P. Pharoah^1^, Clement A. Adebamowo^2,4,5,6^

1. Department of Primary Care and Public Health, University of Cambridge, Cambridge, United Kingdom

2. Institute of Human Virology Nigeria, Abuja, Nigeria

3. Institute for Genome Sciences, Department of Microbiology and Immunology, University of Maryland School of Medicine, Baltimore, Maryland, United States of America

4. Greenebaum Comprehensive Cancer Center, and the Department of Epidemiology, University of Maryland School of Medicine, Baltimore, Maryland, United States of America

5. Center for Bioethics and Research Ibadan, Nigeria

6. Institute of Human Virology, University of Maryland School of Medicine, Baltimore, Maryland, United States of America

Corresponding author

Clement A. Adebamowo MD, ScD; Institute of Human Virology Building; University of Maryland, School of Medicine, 725 West Lombard Street, Baltimore, MD 21201

[cadebamowo@som.umaryland.edu](mailto:cadebamowo@som.umaryland.edu)

**Supplementary Tables**

Supplementary Table 1: Sensitivity analysis examining different cut off points.

|  | Persistent high-risk HPV | | | | |
| --- | --- | --- | --- | --- | --- |
|  | HIV negative | |  | HIV positive | |
|  | OR (95% CI) | *p* |  | OR (95% CI) | *p* |
| *Lactobacillus* dominant |  |  |  |  |  |
| Cut point 50% | 0.44 (0.19 – 1.01) | 0.05 |  | 1.26 (0.71 – 2.23) | 0.42 |
| Cut point 55% | 0.42 (0.19 – 0.93) | 0.03 |  | 1.20 (0.69 – 2.08) | 0.52 |
| Cut point 60% | 0.39 (0.17 – 0.91) | 0.03 |  | 1.41 (0.80 – 2.48) | 0.24 |
| Cut point 65% | 0.41 (0.17 – 0.95) | 0.04 |  | 1.46 (0.84 – 2.53) | 0.18 |
| Cut point 70% | 0.35 (0.14 – 0.89) | 0.03 |  | 1.25 (0.73 – 2.14) | 0.41 |
| Cut point 75% | 0.39 (0.16 – 0.99) | 0.05 |  | 1.21 (0.71 – 2.08) | 0.48 |
| Cut point 80% | 0.50 (0.20 – 1.24) | 0.13 |  | 1.20 (0.69 – 2.07) | 0.52 |
| *L.crispatus* dominant |  |  |  |  |  |
| Cut point 50% | 0.35 (0.08 - 1.53) | 0.16 |  | 1.65 (0.78 – 3.50) | 0.19 |
| Cut point 55% | 0.35 (0.08 – 1.53) | 0.16 |  | 1.79 (0.78 – 4.08) | 0.17 |
| Cut point 60% | 0.38 (0.09 – 1.65) | 0.20 |  | 1.68 (0.72 – 3.94) | 0.23 |
| Cut point 65% | 0.41 (0.09 – 1.80) | 0.24 |  | 0.97 (0.30 – 3.12) | 0.97 |
| Cut point 70% | 0.22 (0.03 – 1.44) | 0.11 |  | 0.97 (0.30 – 3.12) | 0.97 |
| Cut point 75% | 0.22 (0.03 – 1.44) | 0.11 |  | 0.97 (0.30 – 3.12) | 0.97 |
| Cut point 80% | 0.24 (0.04 – 1.62) | 0.14 |  | 0.67 (0.12 – 3.73) | 0.65 |
| *Each model is adjusted for age | | | | | |

Supplementary Table 2: Relationship between missing vaginal microbiota and participant characteristics.

| **Characteristic** | **Missing microbiota - baseline*** | |  | **Missing microbiota – follow up**** | |
| --- | --- | --- | --- | --- | --- |
|  | OR (95% CI) | *p* value |  | OR (95% CI) | *p* value |
| Age, years | 1.01 (0.98 – 1.05) | 0.50 |  | 0.96 (0.89 – 1.03) | 0.26 |
| Socioeconomic status |  |  |  |  |  |
| Low | 1.00 |  |  | 1.00 |  |
| Middle | 0.28 (0.13 – 0.59) | 0.001 |  | 1.68 (0.51 – 5.51) | 0.39 |
| High | 0.95 (0.52 – 1.74) | 0.88 |  | 0.54 (0.06 – 4.84) | 0.59 |
| Education, years |  |  |  |  |  |
| ≤ 6 | 1.00 |  |  |  |  |
| 7 – 12 | 0.51 (0.18 – 1.43) | 0.20 |  | 0.63 (0.10 – 34.00) | 0.62 |
| >12 | 0.64 (0.26 – 1.59) | 0.34 |  | 0.77 (0.15 – 3.84) | 0.75 |
| Menopausal |  |  |  |  |  |
| No | 1.00 |  |  | 1.00 |  |
| Yes | 1.62 (0.74 – 3.55) | 0.22 |  | 1.03 (0.22 – 4.93) | 0.97 |
| Time since last menstrual period, days |  |  |  |  |  |
| ≤15 | 1.00 |  |  | 1.00 |  |
| >15 | 0.75 (0.37 – 1.54) | 0.44 |  | - | - |
| Douche regularly |  |  |  |  |  |
| No | 1.00 |  |  | 1.00 |  |
| Yes | 2.03 (0.43 – 9.64) | 0.37 |  | 1.38 (0.15 – 12.48)) | 0.77 |
| Body Mass Index, kg/m2 |  |  |  |  |  |
| Normal weight, 18.5 – 24.9 | 1.00 |  |  | 1.00 |  |
| Overweight, 25.0 – 29.9 | 0.62 (0.29 – 1.35) | 0.23 |  | 2.45 (0.48 – 12.59) | 0.43 |
| Obese, ≥ 30.0 | 1.13 (0.51 – 2.51) | 0.77 |  | 2.57 (0.45 – 14.62) | 0.19 |
| HIV status |  |  |  |  |  |
| Negative | 1.00 |  |  | 1.00 |  |
| Positive | 1.30 (0.67 – 2.53) | 0.77 |  | 0.03 (0.00 – 0.27) | 0.001 |
| Sex within 24 hours |  |  |  |  |  |
| No | 1.00 |  |  | 1.00 |  |
| Yes | 0.41 (0.12 – 1.43) | 0.16 |  | 1.13 (0.24 – 5.42) | 0.81 |
| Total sex partners in 1 year | 0.70 (0.40 – 1.30) | 0.28 |  | 1.33 (0.61 – 2.90) | 0.47 |
| Condom Use |  |  |  |  |  |
| No | 1.00 |  |  | 1.00 |  |
| Yes | 5.70 (0.51 – 64.17) | 0.16 |  | 2.25 (0.26 – 19.81) | 0.47 |
| Oral contraceptive use |  |  |  |  |  |
| No | 1.00 |  |  | 1.00 |  |
| Yes | 0.80 (0.28 – 2.27) | 0.83 |  | 0.70 (0.09 – 5.68) | 0.74 |
| Persistent hrHPV  No  Yes | 1.00  0.51 (0.25 – 1.02) | 0.06 |  | 1.00  0.33 (0.07 – 1.57) | 0.17 |
| *Number of missing microbiota results at baseline = 56  **Number of missing microbiota results at follow up = 13 | | | | | |

Supplementary Table 3: Effect estimates for the association between the vaginal microbiota and persistent hrHPV obtained using imputed datasets.

|  | Persistent high-risk HPV | | | | |
| --- | --- | --- | --- | --- | --- |
|  | HIV negative | |  | HIV positive | |
|  | OR (95% CI) | *p* |  | OR (95% CI) | *p* |
| Model 1* |  |  |  |  |  |
| *Lactobacillus* dominant microbiota |  |  |  |  |  |
| <70% | 1.00 |  |  | 1.00 |  |
| ≥70% | 0.33 (0.14 – 0.81) | 0.02 |  | 1.31 (0.78 – 2.23) | 0.30 |
| Model 2* |  |  |  |  |  |
| *L.crispatus* dominant microbiota |  |  |  |  |  |
| <70% | 1.00 |  |  | 1.00 |  |
| ≥70% | 0.27 (0.04 – 1.70) | 0.16 |  | 1.26 (0.38 – 4.16) | 0.70 |
|  |  |  |  |  |  |
| Model 3* |  |  |  |  |  |
| Community State Assignment |  |  |  |  |  |
| CST IV-B | 1.00 |  |  | 1.00 |  |
| CST I-B | - |  |  | 0.95 (0.50 – 1.82) | 0.88 |
| CST III | 0.67 (0.28 – 1.61) | 0.38 |  | 1.03 (0.39 – 2.70) | 0.95 |
| CST II | 0.28 (0.04– 2.02) | 0.21 |  | 1.01 (0.40 – 2.55) | 0.99 |
| CST I | 0.29 (0.06 – 1.46) | 0.13 |  | 1.30 (0.54 – 3.10) | 0.56 |
| *Each model is adjusted for age | | | | | |

Supplementary Table 4: Association between potential risk factors and the vaginal microbiome ignoring within individual clustering of responses

| **Characteristic** | ***Lactobacillus* dominant microbiota*** | |  | ***L. crispatus* dominant microbiota**** | |
| --- | --- | --- | --- | --- | --- |
|  | OR (95% CI) | *p* value |  | OR (95% CI) | *p* value |
| Age, years | 1.00 (0.98 – 1.03) | 0.91 |  | 1.01 (0.97 – 1.06) | 0.57 |
| Socioeconomic status |  |  |  |  |  |
| Low | 1.00 |  |  | 1.00 |  |
| Middle | 0.92 (0.58 – 1.45) | 0.72 |  | 1.03 (0.42 – 2.55) | 0.95 |
| High | 0.95 (0.52 – 1.74) | 0.88 |  | 1.29 (0.42 – 3.94) | 0.66 |
| Education, years |  |  |  |  |  |
| ≤ 6 | 1.00 |  |  |  |  |
| 7 – 12 | 1.42 (0.67 – 3.02) | 0.36 |  | 0.57 (0.09 – 3.56) | 0.55 |
| >12 | 1.76 (0.87 – 3.53) | 0.11 |  | 1.91 (0.43 – 8.51) | 0.40 |
| Menopausal |  |  |  |  |  |
| No | 1.00 |  |  | 1.00 |  |
| Yes | 1.28 (0.70 – 2.37) | 0.43 |  | 1.89 (0.66 – 5.40) | 0.23 |
| Time since last menstrual period, days |  |  |  |  |  |
| ≤15 | 1.00 |  |  | 1.00 |  |
| >15 | 1.08 (0.65 – 1.78) | 0.77 |  | 1.29 (0.43 – 3.83) | 0.65 |
| Douche regularly |  |  |  |  |  |
| No | 1.00 |  |  | 1.00 |  |
| Yes | 1.23 (0.78 – 1.95) | 0.37 |  | 0.90 (0.36 – 2.22) | 0.82 |
| Body Mass Index, kg/m2 |  |  |  |  |  |
| Normal weight, 18.5 – 24.9 | 1.00 |  |  | 1.00 |  |
| Overweight, 25.0 – 29.9 | 1.21 (0.73 – 2.00) | 0.45 |  | 1.57 (0.52 – 4.72) | 0.43 |
| Obese, ≥ 30.0 | 0.93 (0.53 – 1.63) | 0.79 |  | 2.15 (0.70 – 6.84) | 0.19 |
| Vaginal pH |  |  |  |  |  |
| <4.5 | 1.00 |  |  | 1.00 |  |
| 4.5 – 5.5 | 0.70 (0.21 – 2.33) | 0.56 |  | 1.34 (0.48 – 3.77) | 0.58 |
| >5.5 | 0.84 (0.28 – 2.58) | 0.77 |  | - |  |
| HIV status |  |  |  |  |  |
| Negative | 1.00 |  |  | 1.00 |  |
| Positive | 0.53 (0.34 – 0.84) | 0.007 |  | 0.33 (0.14 – 0.74) | 0.008 |
| Sex within 24 hours |  |  |  |  |  |
| No | 1.00 |  |  | 1.00 |  |
| Yes | 1.08 (0.61 – 1.90) | 0.79 |  | 0.69 (0.20 – 2.40) | 0.56 |
| Total sex partners in lifetime | 0.98 (0.92 – 1.04) | 0.46 |  | 0.98 (0.86 – 1.11) | 0.72 |
| Total sex partners in 1 year | 1.04 (0.72 – 1.51) | 0.83 |  | 0.71 (0.31 – 1.65) | 0.43 |
| Condom Use |  |  |  |  |  |
| No | 1.00 |  |  | 1.00 |  |
| Yes | 0.78 (0.51 – 1.19) | 0.25 |  | 0.65 (0.28 – 1.52) | 0.32 |
| Oral contraceptive use |  |  |  |  |  |
| No | 1.00 |  |  | 1.00 |  |
| Yes | 0.91 (0.44 – 1.93) | 0.83 |  | 0.33 (0.05 – 2.56) | 0.30 |
| *Relative abundance of *Lactobacillus* species > 70%  **Relative abundance of *L. crispatus* > 70% | | | | | |

Supplementary Table 5: Association between the vaginal microbiota and persistent hrHPV ignoring within person correlation of covariates.

|  | Persistent high-risk HPV | | | | |
| --- | --- | --- | --- | --- | --- |
|  | HIV negative | |  | HIV positive | |
|  | OR (95% CI) | *p* |  | OR (95% CI) | *p* |
| Model 1* |  |  |  |  |  |
| *Lactobacillus* dominant microbiota |  |  |  |  |  |
| <70% | 1.00 |  |  | 1.00 |  |
| ≥70% | 0.35 (0.14 – 0.91) | 0.03 |  | 1.25 (0.75 – 2.09) | 0.39 |
| Model 2* |  |  |  |  |  |
| *L.crispatus* dominant microbiota |  |  |  |  |  |
| <70% | 1.00 |  |  | 1.00 |  |
| ≥70% | 0.22 (0.03 – 1.80) | 0.16 |  | 1.15 (0.34 – 3.87) | 0.83 |
|  |  |  |  |  |  |
| Model 3* |  |  |  |  |  |
| Community State Assignment |  |  |  |  |  |
| CST IV-B | 1.00 |  |  | 1.00 |  |
| CST I-B | - |  |  | 0.95 (0.51 – 1.77) | 0.88 |
| CST III | 0.67 (0.23 – 1.94) | 0.47 |  | 1.03 (0.39 – 2.70) | 0.95 |
| CST II | 0.28 (0.03 – 2.51) | 0.25 |  | 1.01 (0.40 – 2.53) | 0.99 |
| CST I | 0.29 (0.06 – 1.45) | 0.13 |  | 1.30 (0.57 – 2.95) | 0.54 |
| *Each model is adjusted for age | | | | | |
